# Sociodemographic and lifestyle determinants of blood pressure in adult Nigerians

**DOI:** 10.1093/inthealth/ihaf048

**Published:** 2025-05-02

**Authors:** Oluseyi Adegoke, Ifedayo A Odeniyi, Oluwadamilola O Ojo, Obianuju B Ozoh, Ayesha O Akinkugbe, Njideka U Okubadejo

**Affiliations:** Department of Medicine, Faculty of Clinical Sciences, College of Medicine, University of Lagos, P.M.B. 12003 Idi-Araba, Lagos State, Nigeria; Department of Medicine, Lagos University Teaching Hospital, Idi-Araba, Lagos State, Nigeria; Department of Medicine, Faculty of Clinical Sciences, College of Medicine, University of Lagos, P.M.B. 12003 Idi-Araba, Lagos State, Nigeria; Department of Medicine, Lagos University Teaching Hospital, Idi-Araba, Lagos State, Nigeria; Department of Medicine, Faculty of Clinical Sciences, College of Medicine, University of Lagos, P.M.B. 12003 Idi-Araba, Lagos State, Nigeria; Department of Medicine, Lagos University Teaching Hospital, Idi-Araba, Lagos State, Nigeria; Department of Medicine, Faculty of Clinical Sciences, College of Medicine, University of Lagos, P.M.B. 12003 Idi-Araba, Lagos State, Nigeria; Department of Medicine, Lagos University Teaching Hospital, Idi-Araba, Lagos State, Nigeria; Department of Medicine, Faculty of Clinical Sciences, College of Medicine, University of Lagos, P.M.B. 12003 Idi-Araba, Lagos State, Nigeria; Department of Medicine, Lagos University Teaching Hospital, Idi-Araba, Lagos State, Nigeria; Department of Medicine, Faculty of Clinical Sciences, College of Medicine, University of Lagos, P.M.B. 12003 Idi-Araba, Lagos State, Nigeria; Department of Medicine, Lagos University Teaching Hospital, Idi-Araba, Lagos State, Nigeria

**Keywords:** blood pressure, hypertension, lifestyle determinants, Nigeria, public health interventions, sociodemographic determinants

## Abstract

**Background:**

Blood pressure (BP) trends are influenced by genetic, sociodemographic and lifestyle factors, with notable population-specific variations. This study assessed the impact of these determinants on BP in urban Nigerians to identify high-risk subgroups and inform targeted interventions.

**Methods:**

We conducted a cross-sectional study of 5076 adults ages 18–92 y (51.1% female) from Lagos, Nigeria. Data were collected using a modified World Health Organization STEPS protocol, including standardized BP measurements. Multiple linear regression models evaluated the effects of sociodemographic (age, sex, education, marital status and occupation) and lifestyle factors (tobacco/alcohol use, physical activity and fruit/vegetable consumption) on systolic BP (SBP) and diastolic BP (DBP) trends.

**Results:**

Age and marital status (married/cohabiting) significantly predicted higher SBP and DBP. Male sex, previously married, low physical activity and current tobacco use independently predicted elevated SBP, while alcohol consumption and employment type (salaried and self-employment) predicted higher DBP. The impacts of these factors on SBP and DBP ranged from β=0.03 to 0.28. Low fruit/vegetable consumption was not a significant independent predictor.

**Conclusions:**

Sociodemographic and lifestyle factors exhibit unique patterns in influencing BP among urban Nigerians. Tailored public health strategies, including alcohol/tobacco risk awareness, access to health screening and socio-economic/marital support, are essential for effective hypertension prevention in this population.

## Introduction

Blood pressure (BP) is influenced by both genetic and environmental factors. Ethnic and geographic disparities in the prevalence and magnitude of hypertension within and across continents may be driven by multiple factors that have not been clearly elucidated.^1,2^ There is evidence to suggest that an increase in BP with age is mediated, to some degree, by lifestyle.^3–5^ This position is further strengthened by studies in secluded populations that have not found a consistent trend in an increase in BP with advancing age.^6,7^

In high-income countries, an inverse association between socio-economic status (SES) and mean BP has been reported.^8^ This association holds true for most markers of SES, including income, occupation and level of education. In sub-Saharan Africa (SSA), the pattern of association between SES and BP is more diverse and variable.^9^ A longitudinal study from South Africa reported that higher education and income were independently associated with higher diastolic BP (DBP) in men, but with lower systolic BP (SBP) and DBP in women.^10^ In contrast, a study from Senegal reported illiteracy, occupational category and working conditions were factors associated with elevated BP.^11^

The influence of SES, demographics and lifestyle on the BP of Nigerians has not been well described. Most of the data regarding the sociodemographic and lifestyle characteristics that influence BP among adults are extrapolated from studies conducted in other populations. Understanding the role of these factors in our population will both identify subgroups at increased risk of elevated BP and provide targets for public health interventions to reduce the burden. This study was designed to evaluate the contribution of selected sociodemographic and lifestyle determinants on the BP profiles of adults in Nigeria.

## Methods

### Study design and study site

We utilized data collected from our cross-sectional, population-based hypertension survey conducted in the densely populated urban area of Lagos State, Nigeria, the detailed methodology of which has been previously described.^12^ Lagos is the commercial capital of Nigeria, with a population of about 14 million at the time of this study, which was approximately 10% of the national population. Lagos has a population density of a little over 18 000/km^2^ and is one of the fastest growing cities in the world.^12,13^ A minimum sample size of 1287 was calculated for the survey based on a previous meta-analysis of the prevalence of hypertension in urban areas of Nigeria of 30.6%, with 99% confidence level.^14^ The survey recruited 5365 participants. Of these, only 5076 participants had complete sociodemographic and lifestyle data and were included in this analysis.

### Study participants

The initial recruitment included individuals ≥16 y of age residing in households within the community and excluded institutionalized populations living in prisons, hospitals, school dormitories and nursing homes.

### Recruitment

A stratified multistage random sampling approach was used to select eligible participants from selected households over an 8-month period (May–December 2017). From the total of 16 urban densely populated mixed-income local government areas (LGAs) in metropolitan Lagos, 8 LGAs were randomly selected: Ikeja, Apapa, Mushin, Agege, Lagos Island, Lagos Mainland, Ifako-Ijaye and Oshodi-Isolo. Using enumeration areas (EAs) from the 1991 population census as secondary sampling units, four EAs were randomly selected per LGA. With the aid of the official map from the National Population Commission for each EA, households were randomly selected. The number of households selected from each EA per LGA was dependent on the calculated proportion required from that EA to contribute to the total sample size, based on the documented population size of each LGA and density of each EA. On average, a minimum of 200 households were selected per EA. Each selected household was the tertiary sampling unit from which consenting participants who met the inclusion criteria were recruited.

### Data collection

Trained interviewers who were experienced field workers conducted in-person interviews using a modified World Health Organization (WHO) STEPS protocol and conducted BP measurements using a standard protocol.^12,15^ The questionnaire was piloted in a non-participating LGA and standardized prior to study commencement. Sociodemographic and lifestyle data obtained included age, sex, highest level of education, marital status, occupation, tobacco and alcohol use, level of physical activity and intake of fruits and vegetables. We quantified frequency and level of physical activity as ‘recommended’ if they engaged in moderate physical activity lasting for >2.5 h/week or vigorous physical activity lasting >1 h 15 min/week. ‘Low or no physical activity’ was recorded if physical activity was less than recommended or there was none at all based on the American Heart Association recommendation on physical activity in adults.^16^ The recommended daily intake of fruits and vegetables was calculated as 2 cups of fruit and 2.5 cups of vegetables.^15^ Self-reported pre-existing diagnosis of hypertension was documented. BP was measured using an Omron sphygmomanometer (with appropriate cuff sizes) that was calibrated before the first use and at the beginning of every week thereafter. BP measurements were conducted with the participants seated and rested, three times (1–3 min apart) with documentation of the average of the last two readings only.^12,13^

### Data management and statistical analysis

Data were analysed using the Statistical Package for Social Sciences (SPSS) version 20 (IBM, Armonk, NY, USA). We conducted an initial diagnostic check on our dataset using histograms and Q-Q plots. Variance inflation factor (VIF) analysis was performed to check for multicollinearity among the independent variables. Age (in years), SBP and DBP are presented as mean±standard deviation (SD) and comparison between groups was done using the Student's t-test. Marital status, level of education, occupation, tobacco use, alcohol use and fruits and vegetables consumption are presented as frequencies (percentages) and intergroup comparisons were done using χ^2^ analysis. Multiple linear regression analysis models were used to test if marital status, occupation, tobacco use, alcohol use and fruits and vegetables consumption significantly predicted SBP and DBP, with and without controlling for age and sex. Level of education was excluded from the models, as it demonstrated moderate collinearity (VIF threshold 3.6) with the other independent variables. The level of statistical significance was set at p < 0.05.

## Results

The study included 5076 participants, comprising 2594 females (51.1%) and 2481 males (48.9%), with an age range of 18–92 y. The majority (89.0%) of the population were 20–59 y of age.

### Basic characteristics and lifestyle determinants

Table 1 shows the basic characteristics and lifestyle determinants of the study population stratified by sex. Both sexes were comparable in age.

**Table 1.** Basic sociodemographic and lifestyle determinants of the study population stratified by sex

Independent variables	Total (N = 5075)	Females (n = 2594)	Males (n = 2481)
Age (years), mean ± SD	36.8 ± 12.6	36.5 ± 12.8	37.0 ± 12.3
SBP (mmHg), mean ± SD	125.5 ± 17.2	123.1 ± 17.7	128.0 ± 16.2*
DBP (mmHg), mean ± SD	79.8 ± 12.6	79.5 ± 12.6	80.1 ± 12.6
Highest level of education, n (%)			
No formal education	183 (3.6)	120 (4.6)	63 (2.5)*
Completed primary	263 (5.2)	142 (5.5)	121 (4.9)
Completed secondary	3444 (67.9)	1710 (65.9)	1734 (69.9)
Post-secondary	1185 (23.3)	622 (24.0)	563 (22.7)
Marital status, n (%)			
Never married	1532 (30.2)	596 (23.0)	936 (37.7)*
Married/cohabiting	3355 (66.1)	1849 (71.3)	1506 (60.7)
Previously married	188 (3.7)	149 (5.7)	39 (1.6)
Occupation, n (%)			
Unemployed	254 (5.0)	117 (4.5)	137 (5.5)*
Homemaker	50 (1.0)	49 (1.9)	1 (< 0.01)
Teacher	340 (6.7)	179 (6.9)	161 (6.5)
Salaried employment	360 (7.1)	144 (5.6)	216 (8.7)
Self-employed	4071 (80.2)	2105 (81.1)	1966 (79.2)
Tobacco use, n (%)			
Never smoked	4620 (91.0)	2556 (98.5)	2064 (83.2)*
Current smoker	316 (6.2)	21 (0.8)	295 (11.9)
Previous smoker	139 (2.7)	17 (0.7)	122 (4.9)
Alcohol use, n (%)			
Never drank	3357 (66.1)	2151 (82.9)	1206 (48.6)*
Current drinker	1265 (24.9)	270 (10.4)	995 (40.1)
Previous drinker	453 (8.9)	173 (6.7)	280 (11.3)
Physical activity, n (%)			
None or low	3982 (78.5)	2207 (85.1)	1775 (71.5)*
Recommended	1093 (21.5)	387 (14.9)	706 (28.5)
Consumption of recommended amount of fruits, n (%)			
Yes	4478 (88.2)	2361 (91.0)	2117 (85.3)*
No	597 (11.8)	233 (9.0)	364 (14.7)
Consumption of recommended amount of vegetables, n (%)			
Yes	3381 (66.6)	1840 (70.9)	1541 (62.1)*
No	1694 (33.4)	754 (29.1)	940 (37.9)

* Statistically significant at p < 0.0001.

About two-thirds (67.9%) of the total population had at least a secondary school education, with more males (1734 [69.9%]) than females (1710 [65.9%]) attaining this educational status (p < 0.0001). Two-thirds (66.1%) of the population were either married or cohabiting, with a higher prevalence in females (1849 [71.3%]) than in males (1506 [60.7%]) (p < 0.0001).

Self-employment was the most common occupation (4071 [80.2%]), with comparable frequencies in males and females. Of those in salaried employment (360 [7.1%]), 106 (29.4%) were employed by the government while 254 (70.6%) were employed in private institutions.

A total of 316 (6.2%) smoked tobacco, with a higher prevalence in males (295 [11.9%]) than females (21 [0.8%]) (p < 0.0001). A significantly higher proportion of females compared with males reported adequate consumption of fruits and vegetables (p < 0.0001). Conversely, a significantly higher proportion of males compared with females practiced the recommended level of physical activity (p < 0.0001). With the exception of no/low physical activity, the poor lifestyle practices occurred significantly more often among males compared with females.

Males had significantly higher mean SBP compared with females (128.0±16.2 vs 123.1±17.7; p < 0.0001), but the mean DBPs were comparable (80.1±12.6 vs 79.5±12.6; p = 0.129).

### Effects of selected sociodemographic and lifestyle determinants on BP

Results of multiple linear regression analysis to examine the predictive effect of marital status, occupation, tobacco use, alcohol use, physical activity and fruits and vegetables consumption on SBP are shown in Table 2. The overall model was statistically significant [F(13, 5061) = 25.079, p ≤ 0.0001], suggesting that the combined predictors significantly explained the variance in SBP. However, the model accounted for a small proportion (R^2^ = 0.06) of the variance in SBP. Currently married/cohabiting, previously married, current tobacco use, current and previous alcohol consumption and low fruits consumption showed significant positive association with SBP (p < 0.05), while low physical activity, previous tobacco use and low consumption of vegetables did not reach statistical significance (p>0.05). Teaching as an occupation showed a significant inverse association with SBP (t = −4.03, p < 0.0001).

**Table 2.** Multiple linear regression of independent variables as predictors of SBP with and without age and sex control

	Without age and sex control^a^		With age and sex control^b^	
Independent variable	β (SE)	β (t-value)	β (SE)	β (t-value)
Constant	120.43 (1.25)	(96.40)	106.71 (1.38)	(77.26)
Age (years)			0.39 (0.02)	0.28 (16.87)*
Sex				
Female			Reference	
Male			4.54 (0.51)	0.13 (8.89)*
Marital status				
Never married	Reference		Reference	
Married/cohabiting	6.04 (0.56)	0.17 (10.71)*	1.36 (0.64)	0.04 (2.13)**
Previously married	14.71 (1.31)	0.162 (11.21)*	4.67 (1.45)	0.05 (3.21)**
Occupation				
Unemployed	Reference		Reference	
Homemakers	−3.03 (2.61)	−0.02 (−1.16)	1.77 (2.52)	0.01 (0.70)
Schooling	−5.69 (1.41)	−0.08 (−4.03)*	−2.42 (1.37)	−0.04 (−1.77)
Salaried employment	−0.658 (1.40)	−0.01 (−0.47)	−0.06 (1.35)	−0.001(−0.05)
Self-employed	−1.655 (1.13)	−0.04 (−1.47)	−0.58 (1.08)	−0.01 (−0.54)
Tobacco use				
Never smoked	Reference		Reference	
Current smoker	3.60 (1.04)	0.05 (3.46)**	2.03 (1.01)	0.03 (2.01)**
Previous smoker	0.04 (1.47)	0.00 (0.03)***	−2.17 (1.42)	−0.02 (−1.53)
Alcohol use				
Never drank	Reference		Reference	
Current drinker	3.03 (0.60)	0.08 (5.09)*	1.05 (0.60)	0.03 (1.75)
Previous drinker	2.69 (0.85)	0.05 (3.15)**	1.07 (0.83)	0.02 (1.30)
Physical activity				
Recommended	Reference		Reference	
None or low	0.83 (0.59)	0.02 (1.41)	1.27 (0.57)	0.03 (2.24)***
Consumption of recommended amount of fruits				
Yes	Reference		Reference	
No	1.562 (0.79)	0.03 (1.99)***	1.45 (0.76)	0.03 (1.92)
Consumption of recommended amount of vegetables				
Yes	Reference		Reference	
No	0.75 (0.54)	0.02 (1.38)	0.69 (0.52)	0.02 (1.32)

SE: standard error.

a Model R^2^ = 0.06, adjusted R^2^ = 0.06, F(13, 5061) = 25.079, p≤0.0001.

b Model R^2^ = 0.13, adjusted R^2^ = 0.13, F(15, 5059) = 51.001, p ≤ 0.0001.

*Statistically significant at *p < 0.0001, **p < 0.01 and ***p < 0.05.

The individual impact of the predictors on SBP were generally low, with currently married/cohabiting having the strongest positive impact (β = 0.17), while low consumption of fruits had the least (β = 0.03).

When age and sex were added to the model (Table 2), it remained statistically significant [F(15, 5059) = 51.001, p ≤ 0.0001] and the overall model accounted for a greater proportion (R^2^ = 0.13) of the variance in SBP than when age and sex were excluded. The results showed that age (t = 16.87, p < 0.0001), male sex (t = 8.889, p < 0.0001), currently married/cohabiting (t = 2.13, p = 0.033), previously married (t = 3.21, p = 0.001), current tobacco use (t = 2.01, p = 0.044) and no/low physical activity (t = 2.27, p = 0.025) were statistically significant independent positive predictors of SBP. Occupation, alcohol use and fruits consumption showed no statistically significant relationship with SBP, independent of age and sex (p > 0.05).

For each 1-y increase in age, there was an increase of 0.39 mmHg in SBP. Compared with the never married, currently married/cohabiting or previously married were associated with a 1.36 mmHg and 4.67 mmHg increase in SBP, respectively.

Thus the regression equation for predicting SBP is 106.71 + 0.39* (age) + 4.54* (sex) + 1.36* (married/cohabiting) + 4.67* (previously married) + 2.03*(current tobacco use) + 1.27* (no/low physical activity).

With respect to their impact sizes on SBP variability, age had the strongest positive impact on SBP (β = 0.28), followed by male sex (β = 0.13), while the impacts of current tobacco use (β=0.03) and no/low physical activity (β = 0.03) were low. The individual impacts of currently married/cohabiting (β = 0.04) and previously married (β = 0.05) were also observed to reduce in the context of age and sex control.

Table 3 shows the results of multiple linear regression analysis to examine the predictive effect of marital status, occupation, tobacco use, alcohol use, physical activity and fruits and vegetables consumption on DBP with and without age and sex control. The overall models for both regression analyses with [F(15, 5059) = 48.667, p ≤ 0.0001] and without [F(13, 5061) = 33.143, p ≤ 0.0001] age and sex control were statistically significant. However, the model that included age and sex explained a greater proportion of the variance in DBP than did the model without age and sex (R^2^ = 0.13 vs 0.08).

**Table 3.** Multiple linear regression of independent variables as predictors of DBP with and without age and sex control

	Without age and sex control^a^		With age and sex control^b^	
Independent variables	β (SE)	β (t-value)	β (SE)	β (t-value)
Constant	73.10 (0.91)	(80.66)*	65.11 (1.02)	(64.17)*
Age (years)			0.28 (0.02)	0.28 (16.35) *
Sex				
Female			Reference	
Male			0.25 (0.38)	0.01(0.66)
Marital status				
Never married	Reference		Reference	
Married/cohabiting	5.98 (0.41)	0.23 (14.63)*	2.24 (0.47)	0.08 (4.79)*
Previously married	7.50 (0.95)	0.11 (7.88)*	−0.60 (1.07)	−0.01(−0.56)
Occupation				
Unemployed	Reference		Reference	
Homemaker	−1.49 (1.89)	−0.01 (−0.79)	0.71 (1.85)	0.01 (0.39)
Teacher	−2.71 (1.02)	−0.05 (−2.65)**	−071 (1.00)	−0.01 (−0.71)
Salaried employment	2.38 (1.02)	0.05 (2.34)***	2.90 (0.99)	0.06 (2.93)**
Self-employed	1.63 (0.05)	0.05 (2.00)***	2.30 (0.80)	0.07 (2.89)**
Tobacco use				
Never smoked	Reference		Reference	
Current smoker	0.05 (0.75)	0.001 (0.07)	−0.33 (0.74)	−0.01 (−0.44)
Previous smoker	0.84 (1.07)	0.01 (0.78)	−0.02 (1.04)	0.00 (−0.02)
Alcohol use				
Never drank	Reference		Reference	
Current drinker	1.81 (0.43)	0.06 (4.18)***	1.38 (0.44)	0.05 (3.15)**
Previous drinker	1.85 (0.62)	0.04 (2.99)**	1.32 (0.61)	0.03 (2.17)***
Physical activity				
None or low	Reference		Reference	
Recommended	0.72 (0.43)	0.02 (1.69)	0.63 (0.42)	0.92 (1.51)
Consumption of recommended amount of fruits				
Yes	Reference		Reference	
No	−0.87 (0.57)	−0.02 (−1.52)	−0.74 (0.56)	−0.02 (−1.33)
Consumption of recommended amount of vegetables				
Yes	Reference		Reference	
No	0.20 (0.39)	0.01 (0.52)	0.20 (0.38)	0.01 (0.53)

a Model R^2^ = 0.08, adjusted R^2^ = 0.08, F(13, 5061) = 33.143, p ≤ 0.0001.

b Model R^2^ = 0.13, adjusted R^2^ = 0.12, F(15, 5059) = 48.667, p ≤ 0.0001.

Statistically significant at *p < 0.0001, **p < 0.01 and ***p < 0.05.

Age (t = 16.35, p < 0.0001) was a statistically significant positive predictor of DBP, while sex (t = 0.66, p < 0.512) was not. Currently married/cohabiting, salaried employment, self-employment and current and previous alcohol consumption had a significant positive relationship with DBP with and without age and sex controls (p < 0.05). Previously married and teaching as an occupation did not show a statistically significant relationship with DBP independent of age and sex (p > 0.05). Tobacco use, physical activity and fruits and vegetables consumption showed no statistically significant relationship with DBP with or without age and sex controls (p > 0.05).

Age was associated with a 0.28 mmHg increase in DBP for every year increase. Compared with never married, currently married/cohabiting was associated with a 2.24 mmHg increase in DBP, while current alcohol drinker had a 1.38 mmHg increase in DBP compared with never drinkers. Thus the regression equation for predicting DBP is 65.11 + 0.28*(age) + 2.24* (married/cohabiting) + 2.90*(salaried employment) + 2.30*(self-employment) + 1.38*(current alcohol drinker) + 1.32* (previous alcohol drinker).

With respect to their impact on DBP variability, age had the greatest impact (β = 0.276) on DBP. With age and sex control, we observed a reduction in the impact on DBP for currently married/cohabiting (β = 0.23–0.08), current alcohol consumption (β = 0.06–0.05) and previous alcohol consumption (β = 0.04–0.03), while a slight increase in impact was observed for salaried employment (β = 0.05–0.06) and self-employment (β = 0.05–0.07).

Age and currently married/cohabiting were the statistically significant independent positive predictors of both SBP and DBP (Tables 2 and 3).

## Discussion

Our goal was to determine the impact of selected sociodemographic factors and lifestyle practices on the BP profile of urban-dwelling adults in Nigeria. With the high prevalence of hypertension in Nigeria, identifying subgroups at risk of elevated BP or hypertension is critical in order to provide targets for public health interventions.

Our study showed that age, male sex, currently married/cohabiting, previously married, salaried and self-employment, current tobacco use, alcohol use and low physical activity were independent positive predictors of BP. These factors are consistent with those that have been traditionally associated with hypertension and have been part of hypertension risk prediction models in different populations across African, Asian and Western countries and these factors were included in various combinations in those studies.^17–22^ However, these factors accounted for a small proportion of the overall variability observed in BP trends, suggesting that other factors also contribute to BP trends in these populations. This finding is consistent with the multifactorial aetiology of hypertension.^23^

The differing degrees of impact (very low to modest) of these factors on BP are similar to those described in many models used to develop hypertension risk prediction frameworks in many populations.^17,19,21,22^

Our finding that age had the greatest impact on both SBP and DBP and modulated the impacts of the other factors on BP is consistent with global trends where the incidence of hypertension increases with age, reflecting the cumulative effects of vascular aging.^24–27^ Age features consistently with modest impact in virtually all hypertension risk prediction frameworks.^18–22^

The ability of male sex to predict SBP (with modest impact) but not DBP is consistent with the reported higher risk of systolic hypertension in men and its identification as an important hypertension risk predictor in many populations.^18,19,28^ In a hypertension risk framework from Canada, sex was not a significant predictor despite a higher proportion of the men being hypertensive.^20^ However, that study did not differentiate between SBP and DBP and the majority of the participants in the study were middle-aged, a period when rapid increase in BP is also reported in women.^20,29^

Interestingly, we found that being married or cohabiting independently predicted higher SBP and DBP, albeit with very modest impact. This finding is similar to that reported in risk prediction models from some African and Indian communities with population characteristics similar to ours (also with very modest impact in all), but contrasts with the no association reported in a Western country, and it was not included at all in some models.^17–22,30^ The reported association of marital status with BP varied widely with sex in other different populations.^25,31–33^ Marriage is often associated with increased responsibilities, which is often not matched by socio-economic capability, thus leading to pressure and stress, triggering an increase in BP. The reported wide variation may reflect differences in these socio-economic responsibilities as well as the availability or otherwise of marital support systems in such populations. Additionally, a greater tendency towards a sedentary lifestyle and obesity have been reported in married individuals, which may contribute to this increased risk of hypertension, especially in cultures that encourage such lifestyles.^34,35^

While employment status was a significant predictor of hypertension in risk prediction models that were mainly from African and Asian countries, its reported impact on BP was generally low.^17,19,22^ In our study, salaried and self-employment status predicted DBP, but with a low impact, a finding similar to that reported in studies from Kenya, China and India, but contrasts with the reduced risk of hypertension reported among self-employed Americans and no risk association among Europeans.^19,36–38^ A possible explanation for these disparities may be differences in the work environment and availability or otherwise of work support systems. It is well documented that an unfavourable work environment and psychosocial work stress factors are associated with an increased risk of hypertension.^39^

The predictive ability of current tobacco use for SBP in our study is consistent with the fact that tobacco smoking increases both local and systemic catecholamine release, which can contribute to vasoconstriction and, in particular, an elevation in SBP.^40^ However, the observed impact of tobacco use on BP in this study was very small, similar to that observed in the hypertension risk prediction models that were developed in African countries.^17,21,22^ In contrast, tobacco use was associated with more modest impacts in the Framingham and China models, with its impact being even greater than that of sex and alcohol in the Chinese study.^18,19^ A possible explanation for this difference in impact sizes may be the relatively lower proportion of tobacco smokers in our study and in the other African studies compared with the studies from temperate countries. However, tobacco use did not predict hypertension risk in the model from Canada.^20^ There are suggestions that inherent genetic and racial/ethnic differences may actually modulate the effect of tobacco smoking on BP, which may partly explain these observed differences.^41,42^

Alcohol causes vagal inhibition and sympathetic activation that lead to an increase in BP and has been associated with the incidence of hypertension.^43^ Although alcohol consumption predicted BP in our study, its impact was relatively small. Alcohol consumption also predicted hypertension risk in models from South Africa, Nigeria, Ethiopia and China, but not in the models from Canada or Framingham.^17–22^ While the reported impact of alcohol in the Nigerian and Ethiopian models were low, they were modest in the models from China and South Africa.^17,19,21,22^ Notwithstanding the seemingly low impact, available evidence suggests that the deleterious effect of alcohol on BP is most pronounced in Africans and that these effects tend to occur even at the lowest levels of alcohol consumption thought to be ‘protective’ in non-African populations.^17,43–45^

Low physical activity promotes weight gain, impairs endothelial function, reduces blood flow and causes arterial stiffness, which may lead to an increase in BP.^46^ In our study, low physical activity significantly predicted SBP, which is consistent with the report that the effect of physical activity is more profound on SBP, although DBP may also be affected.^47^ However, the impact size of low physical activity on BP in our study was low. The reported impact of low physical activity as a predictor of hypertension risk varied widely from very low to modest, while some reported no association at all.^17,19–22,48^ Apart from differences in the sample sizes of these studies, which may have affected the outcome, possible differences in the type of physical activities practiced (which were largely not specified) in the different study populations may partly explain the differences in the impacts observed. Recent evidence suggests that the impact of physical activity on BP may be related to the type of activity practiced (work, commuting or leisure), with commuting or leisure activities being better associated with BP prediction.^49^

Low fruits and vegetables consumption did not predict BP in our study, which is similar to that reported in the hypertension risk prediction model in Canada but contrasts with that from Peru, which reported a significant association with elevated BP.^20,28^ However, the possibility of a type II error cannot be ruled out due to differences in our sample sizes. An inverse relationship between high consumption of fruits/vegetables and BP levels in the Mediterranean population is well documented,^50^ but a meta-analysis found no such relationship consistently in Europe and America.^51^ While it is also acknowledged that a healthy diet is more than fruits and vegetables, studies that incorporated components of a healthy diet beyond fruits and vegetables in their hypertension risk prediction models also varied in their reports from no association to a positive predictive association.^19,22^ Again, there was a marked difference in the sample sizes of these studies, with the study that reported no association having a much smaller sample size, which points to the possibility of type II error. It has been suggested that perhaps it is not just the consumption of a healthy diet that protects, but rather the abstinence from unhealthy ones. A recent study reported a higher risk of hypertension in individuals who continued consumption of foods considered unhealthy despite simultaneous high consumption of fruits, vegetables and other foods considered healthy.^52^

### Clinical implications

Identifying and intervening in sociodemographic and lifestyle factors that promote hypertension risk at the individual level are major pillars in the WHO and the Pan African Society of Cardiology strategies for achieving BP control and a reduction in the burden of hypertension.^53,54^ While the individual impact of the selected sociodemographics and lifestyle factors on BP may appear low or modest, their synergistic effects in accelerating the incidence of hypertension are well documented.^55,56^ This is further supported by the marked reduction in BP levels demonstrated with multipronged interventional strategies targeted at multiple risk factors.^54^ Hence in screening for hypertension risk, the age, sex, marital status, employment status, level of physical activity and alcohol and tobacco use status of the individual are important risk factors that should be assessed and addressed. Screening should commence in early adulthood for all individuals at every healthcare provider encounter well before hypertension develops. This will help identify individuals at risk, enabling individualized early interventions with a subsequent reduction in the incidence of hypertension. However, considering the modest contribution of these factors to the variance in BP, they should be considered in combination with other known risk factors for hypertension (such as family history, dietary sodium, etc) for a complete assessment and intervention.

### Public health implications

This study reflects the complexity of risk factors for hypertension. Further studies are required to fully explore other factors that may also contribute to the BP trend in this population. Meanwhile, our findings highlight the need for early detection and prevention through strategic government policies that address the identified risk factors. First, compulsory health education with emphasis on healthy lifestyles should be included in all school curricula from the primary school level. Second, physical activity should be encouraged by inclusion of mandatory minimum 30-min daily sporting activities into school timetables. Government should encourage walking and cycling as modes of transportation through provision of safe and appropriate pathways for such activities. Third, government should strengthen its socio-economic policies, particularly with reference to providing easily accessible and affordable health screening facilities. The national health insurance scheme (NHIS) should be revived and be more accessible, as only 5% of the Nigerian population have accessed the NHIS, as of 2021, since its inception in 2005.^57^ Provision of affordable and accessible low-cost housing for families as well as access to government-backed low-interest loans may help ameliorate some of the pressures of marital responsibilities. Fourth, standards organizations should enforce health-friendly work environments and the establishment of workplace health systems that incorporate health insurance, periodic education and health screening programs. Fifth, increase taxes on alcohol and tobacco, prohibit their advertisement and restrict their sales to limit access.

### Study limitations

Our study has several limitations. First, it is a cross-sectional study for which one set of data was collected. It lacks longitudinal data, hence causal relationships cannot be inferred. The robust sample size and the population source of our data hopefully attenuate this limitation. Second, the study was conducted in only one state of the country. The advantage of our population source (Lagos) is that it is a multi-ethnic megacity with mixed income and mixed culture, with representation of all the major ethnic groups in the country and lifestyle dynamics typical of urban populations. To the best of our knowledge, this is the first study that specifically explored the predictive ability of selected sociodemographic and lifestyle factors on SBP and DBP trends in Nigeria.

Third, we acknowledge that the use of self-reporting of lifestyle data in this study may be prone to both recall and social desirability bias. We hope that our use of trained interviewers who are experienced field workers as well as the use of a previously standardized questionnaire attenuated this limitation. Our primary data collection did not include laboratory biochemical markers for alcohol and smoking due to funding and logistic challenges. Fourth, data on dietary sodium intake, other dietary patterns, family history, medication use and healthcare system, which may be potential confounding variables, were not collected in the original survey, and we acknowledge that providing this additional insight could have improved the robustness of our data.

### Conclusions

We demonstrated that sociodemographic and lifestyle factors, specifically age, male sex, married and previously married status, low physical activity, salaried and self-employment and tobacco and alcohol use are positive predictors of BP trends in our population. We also demonstrated that these factors show varying degrees of impact on SBP and DBP. Routine screening for these factors from early adulthood could identify individuals who are at increased risk of hypertension and inform prompt individualized intervention strategies, thereby reducing the incidence of hypertension. As an aid to reducing the national burden of hypertension, primordial prevention could be targeted through strategic policies that discourage alcohol and tobacco use, establish workplace health systems, enact socio-economic policies that provide marital support systems and provide infrastructure that encourages walking and cycling as modes of transportation, while continuing health education awareness programs.

## Data Availability

The datasets used and/or analysed during the current study are available from the corresponding author upon reasonable request.
